# An Uncalled for Criticism

**Published:** 1888-01

**Authors:** 


### AN UNCALLED FOR CRITICISM.

The last number of *Correspondenz Blatt für Zahnarzt* contains an editorial article which seems to have been conceived and written in a singular misconception of the leading editorial in this Journal for September last, "Concerning Contests." The editor of *Correspondenz Blatt* does not seem to have the faintest comprehension of the scope and aim of that article. He should know that the editor of this Journal lives more than four hundred miles from the residence of Dr. Bödecker, and that whatever has been published in our editorial pages has been without his knowledge and without any consultation with him. When we said that Dr. Herbst had risen to his present position against the most adverse circumstances, we paid him the highest compliment which we could offer, for Americans honor the man who earns an honorable position far above him who is born to it. We never said that Dr. Bödecker paid Dr. Herbst's expenses to America, and when the editor intimates that we did, and then brands it as a falsehood, he simply exhibits his own disingenuousness. We said that Dr. Bödecker's sacrifices and labors in the establishment, in America, of the Herbst system of filling teeth, simply because he believed that he was serving his profession, should shield him from any charge of ulterior motives, and entitle him to the gratitude of American dentists, even though he might be mistaken concerning the merits of the rotary method of filling teeth. We believe that Dr. Herbst's visit to America resulted in good to American dentistry. There is here a warm feeling of admiration for him as a man, and the attempt of the editor in question to foment an ill-feeling, either toward Herbst in America or Bödecker in Europe, and to place them in an attitude of opposition to each other, is unworthy the journal which he controls. There was nothing in our article that should be offensive to Herbst, and if he is content the editor need not officiously interfere. But if he insists upon standing as the self-elected champion of Herbst, he shall have the field to himself, for we are no man's special advocate.

